# Can you feel the beat? Interoceptive awareness is an interactive function of anxiety- and depression-specific symptom dimensions

**DOI:** 10.1016/j.brat.2010.07.006

**Published:** 2010-11

**Authors:** Barnaby D. Dunn, Iolanta Stefanovitch, Davy Evans, Clare Oliver, Amy Hawkins, Tim Dalgleish

**Affiliations:** a Emotion Research Group, Medical Research Council Cognition and Brain Sciences Unit, 15 Chaucer Road, Cambridge, CB2 7EF, UK; b Sub-department of Clinical Health Psychology, University College London, UK; c Department of Psychology, University of Bath, UK

**Keywords:** Depression, Anxiety, Interoception, Heartbeat perception, Tripartite model

## Abstract

Delineating the differential effects of anxiety versus depression on patterns of information processing has proved challenging. The tripartite model of mood disorders (Clark & Watson, 1991) suggests that one way forward is to adopt a dimensional rather than categorical approach, making it possible to explore the main and interaction effects of depression- and anxiety-specific symptoms on a given cognitive-affective process. Here we examined how the interplay of anxiety-specific arousal and depression-specific anhedonia symptoms in the same individuals relate to interoceptive (bodily) awareness. 113 participants with varying levels of mood disorder symptoms completed a heartbeat perception task to assess interoceptive accuracy. Superior interoception was associated with anxiety-specific arousal symptoms, and this relationship held when controlling for depression-specific anhedonia symptoms and shared general distress symptoms. This main effect was qualified by an interaction between anhedonia and arousal. As anhedonia symptoms increased in severity, the relationship between arousal and interoceptive accuracy became less strong. These results further validate the tripartite framework, help clarify the mixed existing literature on interoception in mood disorders, and suggest that considering the unique and interactive effects of different symptom dimensions is a useful strategy to help identify the cognitive-affective profiles associated with anxiety and depression.

Cognitive approaches to anxiety and depression are underpinned by the proposal that each condition is associated with a relatively distinct pattern of alterations in information processing (Mineka, Rafaeli, & Yovel, 2003), the identification and normalization of which are central to effective differential diagnosis and intervention (e.g. Clark, Beck, & Alford, 1999; Segal et al., 2006). Consequently, there has been a strong research focus on identifying unique cognitive-affective profiles associated with different emotional disorders.

While there is reasonable support for notions of ‘content’ specificity, with anxiety being linked to a preoccupation with threat and depression being associated with loss (for a review see Beck, 2005), compelling evidence of ‘process’ specificity has yet to be established at the diagnostic level. Mixed findings have emerged from studies examining judgment, attentional and mnemonic biases in diagnoses of anxiety versus depression, even when comparing ‘pure’ cases of each condition in the same experiment and on the same measures (Dunn, Stefanovitch, Buchan, Lawrence, & Dalgleish, 2009; Mineka et al., 2003). For example, anxiety disorders have been reliably associated with threat related attentional biases, with an inconsistent picture emerging regarding memory biases. In depression there is robust evidence for preferential recall of negative material but mixed evidence regarding attentional biases (see Williams et al., 1997). It is hard to argue that these information processing biases can fully differentiate between diagnoses of anxiety and depression, given the inconsistent but sometimes positive findings of memory biases in anxiety and attentional biases in depression (see Dunn et al., 2009).

A prototypical candidate for examining processing specificity is that of interoceptive ability. This is the ability to detect changes in the body (including muscles, skin, joints and viscera), enabling individuals to experience bodily ‘feelings’ such as pain, temperature, itch, and hunger (Craig, 2002). There has been increasing theoretical interest in the possibility that interoception may be altered in mood disorders. For example, heightened awareness of bodily arousal, alongside misattributions of the source of the arousal, is believed to contribute to the maintenance of panic disorder and social phobia (e.g. Clark, 1986; Clark & Wells, 1995).

However, empirical support for the differential relationship of anxiety and depression to interoceptive ability presents exactly the sort of mixed picture discussed above. In panic disorder some studies find superior heartbeat perception (e.g. Ehlers & Breuer, 1992; Zoellner & Craske, 1999), but there have been notable failures to replicate this effect and it has been argued that these discrepant findings may reflect the fact that only a subset of panic sufferers show elevated interoception (Van-der-Does, Antony, Ehlers, & Barsky, 2000). Conversely, studies show a non-significant trend for reduced interoceptive accuracy in depression (e.g. Ehlers & Breuer, 1992; Pollatos, Traut-Mattausch, & Schandry, 2009; Van-der-Does, Van-Dyck, & Spinhoven, 1997) and that higher levels of depression symptoms in the general population are associated with less accurate interoception (Pollatos et al., 2009). We recently found that while dysphoric individuals showed impaired interoceptive ability, this deficit normalized in individuals diagnosed with Major Depressive Disorder (Dunn, Dalgleish, Ogilvie, & Lawrence, 2007).

One possible reason for this mixed empirical picture regarding interoceptive performance is that most studies have been underpinned by the current DSM diagnostic classification system. This divides psychological distress into distinct typologies with unique defining features (e.g. “depressed”, “anxious”, or “healthy”) (APA., 1994). However, it is increasingly debated whether such absolute distinctions between diagnoses exist; comorbidity is the rule rather than the exception, individuals often cross diagnostic boundaries over time, and there are ‘boundary disputes’ about how to classify presentations that fall between categories (Mineka, Watson, & Clark, 1998; Widiger & Samuel, 2005).

An alternative to this diagnostic approach is to conceptualize depression and anxiety as dimensional constructs, with individuals lying somewhere on a series of overlapping continua ranging from symptom free to highly symptomatic (e.g. Watson, 2005; Widiger & Samuel, 2005). Elevations on particular dimensions may be specific to a given diagnosis, whereas other dimensions may characterize multiple diagnoses. For example, the tripartite framework (Clark & Watson, 1991; Clark, Watson, & Mineka, 1994) proposes that high levels of ‘general distress’ (non-specific negative affectivity) are a feature of both anxiety and depression. In contrast, specific to anxiety are elevated physiological hyper-arousal, whereas specific to depression are elevated symptoms of anhedonia.^1^

If it is such *dimensions*, rather than diagnoses, that are related to a particular processing style (for example, heightened/diminished interoceptive ability), then this would account for the mixed empirical findings from diagnostic case–control studies. Clinical participants in such studies, despite being diagnostically homogeneous, would most likely be heterogeneous as regards their profile of elevated scores on the pertinent symptom dimensions.

The primary aim of the present study was therefore to examine the influence of the tripartite model symptom dimensions (Watson, 2005) on interoceptive awareness. As well as clarifying a confusing theoretical literature and helping to understand comorbidity, such an approach also has clinical relevance by helping to identify those for whom interoceptive awareness is disturbed. Given that these interoceptive mechanisms may in turn contribute to other cognitive-affective process known to be disturbed in anxiety and depression (cf. Damasio, 1994; Dunn et al., in press; James, 1884; for reviews see Dalgleish, Dunn, & Mobbs, 2009; Dunn, Dalgleish, & Lawrence, 2006), this has the potential to help target body-focused clinical interventions to appropriate individuals (e.g. interoceptive exposure; Ellard et al., 2010).

In formulating our hypotheses, two ways in which dimensions could differentially impact interoceptive ability seemed critical. First, a given dimension could be uniquely associated with interoceptive awareness. Secondly, such main effects could be qualified by interactions; for example, scores on one dimension ‘moderating’ (cf. Baron & Kenny, 1986) the strength of the relationship that exists between interoceptive awareness and scores on another dimension.

Our first hypothesis was that interoceptive accuracy would be greater in individuals with greater arousal, and this would hold when covarying for scores on the other symptom dimensions (cf. Ehlers & Breuer, 1992; Zoellner & Craske, 1999) (i.e. a unique main effect). Second, we predicted that this main effect would be qualified by an interaction between arousal and anhedonia, such that the strength of the relationship observed between interoceptive awareness and arousal would be reduced in those with relatively higher levels of anhedonia symptoms (a moderation relationship).

The motivation for this second hypothesis came from recent findings that a negative relationship between depression and interoception is only found in those with relatively higher trait anxiety (Pollatos et al., 2009). In this study anxiety and depression were not measured as separable dimensions (cf. the tripartite model) but on standard questionnaires (the Spielberger Trait Anxiety Inventory [Spielberger, Gorsuch, Lushene, Vagg, & Jacobs, 1983; STAI-T] and the Beck Depression Inventory [Beck, Ward, Mendelson, Mock, & Erbaugh, 1961; BDI], respectively) and the interactive effect of these questionnaire measures on interoception was only trend significant. Further, it was conducted with healthy undergraduates with generally very low anxiety and depression symptom levels (BDI: mean = 3.49, *SD* = 3.50; STAI-T: mean = 37.74, *SD* = 10.69), so can only provide a hint at what might happen in a sample where the broad ranges of symptoms are included. Nevertheless, these data suggest that in a sample in which wider ranges of anxiety-specific and depression-specific symptoms are measured as separable dimensional constructs, an important interactive effect of these dimensions might emerge.

We measured interoception by asking participants to count the number of heartbeats they felt over varying time intervals (Schandry, 1981) and comparing this to an electrocardiogram (ECG) record (cf. Dunn et al., 2007). Participants completed the short form of the Mood and Anxiety Symptom Questionnaire (MASQ-S) as a measure of the tripartite symptom dimensions (Watson & Clark, 1991; Watson et al., 1995). Appropriate analysis of dimensional influences on cognitive-affective processing requires a different sampling approach to the traditional case–control design prevalent in the literature. Consequently, we recruited a single sample of community volunteers and stratified our recruitment such that the sample represented a wide range of scores on the MASQ dimensions, up to and including clinical levels.

## Method

### Participants

One hundred and thirteen participants (82 women) aged 18–65 years (*M* = 43.09; *SD* = 16.10) were recruited from the Cognition and Brain Science Unit’s database of community volunteers. This holds details of depression and anxiety levels when participants were last contacted, allowing us to stratify recruitment to ensure a broad range of mood disorder symptoms.^2^ Participants were screened using a brief semi-structured interview and excluded if they reported any history of brain injury, psychosis, learning disability, or substance abuse. Fourteen individuals were taking anti-depressant medication (six using SSRIs, three using SNRIs, one using a beta-blocker, and one using a tricyclic). Mean estimated full scale IQ according to the National Adult Reading Test (NART; Nelson, 1982) was 118.45 (*SD* = 7.14). Due to time constraints and because the conceptual focus of the study was on dimensional rather than categorical approaches to the mood disorders, diagnostic status of participants was not recorded. Participants gave written informed consent, were reimbursed the equivalent of US $10 per hour for their time, and the study was approved by the local research ethics committee.

### Symptom measures

On the MASQ-S (Watson & Clark, 1991; Watson et al., 1995), participants are asked to judge for each of 62 items how much they have felt the way described over the past week, ranging from 1 (not at all) to 5 (extremely). Twenty two items measure depression-specific anhedonia symptoms (e.g. “felt like nothing was very enjoyable”, maximum score = 110), 17 items index anxiety-specific hyper-arousal symptoms (e.g. “was trembling or shaking”, maximum score = 85), and 23 items record general distress symptoms common to both anxiety and depression (e.g. “felt sad”; maximum score = 115). Participants additionally completed the BDI-I and STAI-T.

### Interoceptive accuracy

Interoceptive accuracy was measured using the amended version of the Schandry (1981) heartbeat perception task, as described by Ehlers and Breuer (1992). On each of six computerized trials participants counted how many heartbeats they felt over a period of time (2 × 35 s, 2 × 25 s and 2 × 45 s), which was then compared to how many heartbeats were actually measured by ECG. Each trial began with an 800 Hz, 100 ms tone, warning participants to get ready. Three seconds later a 1000 Hz, 50 ms tone was sounded, signalling participants to begin counting their heartbeats. After a variable time period an identical tone was heard, signalling participants to stop counting their heartbeats and to enter their heartbeat estimate. Participants completed one practice heartbeat trial (12 s).

It has been argued that participants may successfully perform this task without any genuine interoception awareness by counting time and then making educated guesses based on beliefs about their heart rate (Ring & Brener, 1996). If this is the case, interoceptive accuracy should be related to time estimation accuracy and resting heart rate (HR) belief accuracy. To explore this possibility, in three time estimation trials (1 × 23 s, 1 × 56 s, 1 × 40 s in length) participants were asked to estimate how many seconds elapsed between two tones. Estimates were then compared to a stopwatch recording. Further, resting HR during a 3 min relaxation period prior to the interoception task was measured and then compared to participants’ estimate of their resting HR to provide a measure of HR belief accuracy. Age, gender, anti-depressant medication status, body mass index (BMI), resting HR, IQ, and physical activity (assessed using the scale described by Ehlers & Breuer, 1992) have also been associated with heartbeat perception accuracy, so these details were additionally measured.

HR was recorded used a BIOPAC™ MP100 unit and an ECG 100B amplifier acquiring data at 200 samples per second, connected to a PC running Acqknowledge 7.2 software (BIOPAC., 1997). Interoception-, time- (on a trial-by-trial basis) and HR belief accuracy are expressed as percentage error scores. These were calculated by taking the modulus of the actual value minus the estimated value, dividing this by the actual value, and then multiplying by 100 ([|actual − estimated| ÷ actual ] x 100) (cf. Ehlers & Breuer, 1992).

### Procedure

Participants were screened and completed the questionnaires. The psychophysiology electrodes were attached, resting HR was recorded, and participants then completed the interoception paradigm, along with other measures not reported here.

## Results

### Exploratory analyses

Table 1 shows mean scores and spread for the measures. There was a satisfactory range of scores on the mood and interoception measures, comparable to previous studies (e.g. Van-der-Does et al., 2000). Of note, 42 participants had a BDI score greater than 10, indicating at least mild levels of depression, and of these 23 participants had a BDI score greater than 15, indicating moderate to severe levels of depression (Shaw, Vallis, & McCabe, 1985). Thirty eight participants had a STAI-T score greater than 50, indicating high levels of trait anxiety. Scores on the MASQ factors also extended into ranges previously found in clinically diagnosed groups (e.g. Pizzagalli, Iosifescu, Hallett, Ratner, & Fava, 2009). This sample is more ‘clinical’ in nature than the one used by Pollatos et al (2009), and in our view is comparable in terms of symptom severity to other stratified sample studies in the literature (e.g. Dunn et al., 2009).

Next, we examined the extent to which interoceptive accuracy was related to potential confound variables. Interoception error score was not significantly associated with age, gender, NART estimated IQ, anti-depressant medication usage, physical activity, or BMI, greatest Pearson’s *r* = .11, *P*s > .20. Interoception error was trend related to resting HR, *r* = .18, *P* = .05, and was significantly related to time estimation error, *r* = .38, *P* < .001, and HR belief error, *r* = .34, *P* < .001. Therefore, these were entered as covariates in subsequent analyses.

### Interoception analyses

Table 2 reports the correlations of each MASQ factor with interoception error (partialling out the nuisance variables). This reveals that higher arousal, but not anhedonia or general distress, is significantly related to greater interoceptive accuracy.

Next, to explore unique and interactive effects of the different MASQ factors on interoceptive accuracy we ran a hierarchical regression. At step 1 we entered the nuisance covariates, at step 2 MASQ-S anhedonia, at Step 3 MASQ-S general distress, at Step 4 MASQ-S arousal, and at step 5 our interaction term, computed as the product of the arousal and anhedonia scores (standardized [Z-scored] to aid interpretation; cf. Baron & Kenny, 1986).

Eight participants’ data were set aside, 2 as they were univariate outliers, 4 as they were multivariate outliers (malhalanobis distance > 24.32 for seven variables; Tabachnik & Fidell, 2001), and 2 as they lacked a valid estimate of HR belief accuracy. Step 1 of the model was significant, *F*(3,101) = 12.50, *P* < .01, Δ*R^2^* = .27. Time estimation error, *t* = 4.10, *P* < .01, *r*_p_ = .38, beta = .35, and HR belief error, *t* = 3.28, *P* < .01, *r_p_* = .31, but not resting HR, *t* < 1, *r*_p_ = .06, beta = .06, were uniquely related to interoception error. Adding anhedonia at Step 2, *F* < 1, and general distress at Step 3, *F* = 1.14, Δ*R^2^* = .01, did not significantly improve model fit. Adding arousal at step 4 of the model did improve fit, *F*(1,98) = 6.90, *P* = .01, Δ*R^2^* = .05, *t* = 2.63, *r_p_* = −.26, beta = −.25, showing that higher interoceptive error is uniquely associated with lower MASQ-S anxious arousal. Adding the interaction term on Step 5 also accounted for a significantly greater proportion of the variance in interoception error, *F*(1,97) = 4.85, *P* = .03, Δ*R^2^* = .03, *t* = 2.20, *r_p_* = .22, beta = .20. The overall model was significant, *F*(7,97) = 7.74, *P* < .001, *R^2^* = .36, a large effect size according to Cohen’s rule of thumb (Cohen, 1988).

**Fig. 1:**
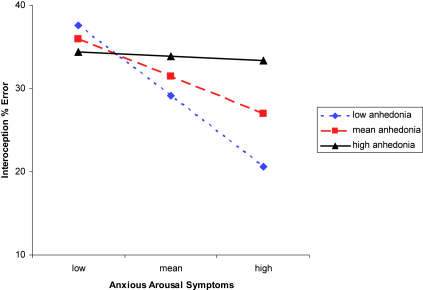
Relationship between MASQ-S anxious arousal and interoception error as a function of MASQ-S anhedonia. Note – Low/High = 1 *SD* below/above the mean. The nuisance variables of resting heart rate, time trial % error, and HR belief accuracy are partialled out before generating this figure.

To illustrate the interaction of arousal and anhedonia on interoception error, we plotted the full regression equation in the recommended way (Aiken & West, 1991) for the mean anhedonia-dimension score and scores plus or minus 1 *SD* (Fig. 1). As anhedonia symptoms increased in severity, the relationship between arousal and interoceptive accuracy became less strong.

We also examined whether this overall pattern was mirrored in analyses which used STAI-T scores to index anxiety and BDI scores to index depression (cf. Pollatos et al., 2009), instead of the MASQ-S. Two participants’ data were excluded as multivariate outliers for this analysis. We found that interoception error was not significantly uniquely related to scores on the STAI-T, *r_p_* = −.09, *P* = .36, nor the BDI, *r_p_* = .14, *P* = .36, suggesting that these measures are less sensitive than the separable dimensions of the MASQ. A significant interaction between BDI and STAI-T was however found, *F*(1,107) = 8.67, *P*<.01, Δ*R^2^* = .07, *t* = 2.94, *r_p_* = .27, beta = .30, that was similar to the Arousal x Anhedonia effect reported above^3^.

## Discussion

The present study examined the interaction of scores on depression-specific anhedonia and anxiety-specific arousal symptom dimensions (as postulated by the tripartite model; Clark & Watson, 1991) on interoceptive accuracy in a large sample of community volunteers with broad ranges of mood disorder symptoms.

Supporting our first hypothesis, anxiety-specific arousal but not the other MASQ-S symptom factors was independently associated with interoception accuracy. Consistent with our second hypothesis, a significant interaction between arousal and anhedonia MASQ-S factors in the prediction of interoceptive accuracy was demonstrated. As anhedonia symptoms increased, the relationship between arousal and interoceptive accuracy became less marked.

These results generally support dimensional models of emotional disorders such as the tripartite framework (Clark & Watson, 1991) and identify different ways in which symptom dimensions can inter-relate. The finding of a significant relationship of arousal with interoception accuracy when controlling for the other MASQ factors is as far as we are aware the first evidence of processing specificity for the anxious arousal dimension. Importantly, no comparable unique relationship emerged between interoception error and general symptom measures (BDI and STAI-T), suggesting that considering theoretically motivated disorder *specific* symptom dimensions may increase explanatory power over and above global severity measures. This complements existing work showing processing specificity of the anhedonia factor on positive information processing (e.g. Dunn et al., 2009; Pizzagalli et al., 2009) and offers construct validity for the anxious arousal dimension. Further, as far as we are aware, the present data is the first demonstration of an interactive relationship between tripartite symptom dimensions and underlying cognitive-affective mechanisms.

The present data help conceptually clarify the currently confusing clinical interoception literature. The subset of individuals with panic disorder who show superior interoception (e.g. Van-der-Does et al., 2000) may be those with few comorbid anhedonia symptoms. Conversely, that we previously found impaired interoception in a mildly depressed community sample and not in a more severely depressed clinical sample (Dunn et al., 2007) may reflect that the clinical group had greater levels of anxious arousal.

Clarifying in which individuals interoception is disturbed may be clinically useful, helping lead to targeted delivery of bodily-focused treatments. For example, the unified transdiagnostic treatment protocol for mood disorders includes modular elements focusing on exposure to interoceptive cues (Ellard et al., 2010). The present data suggest individuals high in anxious arousal symptoms and low in anhedonia may maximally benefit from this particular intervention element.

Here we purposefully eschewed the traditional case–control approach in favour of examining a single community sample selected to represent the range of scores on the tripartite symptom dimensions up to and including the clinical range. Although this design provides the optimal test of the validity of using dimensional approaches to understand profiles of cognitive-affective processing, it would now be interesting to directly contrast the ability of dimensional and diagnostic approaches to model the variance in, for example, interoceptive accuracy, within the same study.

Future research could also explore how different parts of the bodily feedback system are related to particular mood disorders. It can be speculated that some symptoms or disorders lead to elevated or diminished *responses* in the body, whereas other are associated with better or worse *perception* of these bodily changes, and yet others lead to different *appraisals* of the significance of these changes. For example, it is conceivable that PTSD would be linked to elevated bodily responsiveness, whereas panic would be linked to elevated interoception and altered appraisal.

The present study suffers from several limitations. First, we have focused here on cardiac perception and different findings may emerge if other forms of interoception are examined. Second, the effect sizes for the main and interactive effects of the MASQ-S factors were individually small in magnitude (Cohen, 1988). That said, the effect size for the overall model was large (Cohen, 1988), indicating that the tripartite dimensions can account for substantive variance in interoceptive processing. Third, socioeconomic status or ethnicity is not measured, but as far as we are aware these have not previously been associated with interoceptive accuracy.

In summary, the present study suggests that interoceptive accuracy is in part determined by the interaction between scores on depression and anxiety-specific symptom dimensions within the same individual. These findings offer support for dimensional models of affective disorders such as the tripartite framework (Clark & Watson, 1991) and suggest that exploring independent and interactive effects of specific symptom dimensions on cognitive-affective processing is a fruitful avenue for future research. In addition, the results help clarify the confusing literature on interoceptive awareness in emotional disorders and suggest that bodily feedback mechanisms may be a legitimate target for clinical intervention.

## Figures and Tables

**Table 1:** Descriptive statistics for the mood and interoception measures (*N* = 113).

	Mean	*SD*	Min	Max
Interoception % error	32.34	16.93	3.65	85.22
MASQ-S – general distress	41.12	13.06	23	75
MASQ-S – anxious arousal	22.79	5.59	17	48
MASQ-S – anhedonia	58.26	15.34	29	95
MASQ-S – total score	122.16	29.25	70	186
BDI-I	8.82	7.12	0	35
STAI-Trait	43.73	10.18	20	67
STAI-State	37.52	9.06	20	59
Time % error	27.39	16.73	0	92.18
Resting heart rate	71.93	9.25	52.09	94.10
Heart rate belief % error	16.86	27.67	−102.97	100
Physical activity	2.70	1.05	0	6
Body mass index	23.86	4.15	15.80	40.67

Note – BDI-I = Beck Depression Inventory-I; STAI = Spielberger State Trait Anxiety Inventory; MASQ-S = Mood and Anxiety Symptom Questionnaire – Short Form.

**Table 2:** Inter correlations between interoception error and MASQ-S factors.

	1	2	3
Heartbeat % error (1)	–		
MASQ-S anhedonia (2)	*r*_p_ = −.00	–	
MASQ-S general distress (3)	*r*_p_ = −.08	*r*_p_ = .70*	–
MASQ-S anxious arousal (4)	*r*_p_ = −.27*	*r*_p_ = .27*	*r*_p_ = .48*

Note – The nuisance variables of resting heart rate, time trial % error, and HR belief accuracy are partialled out in the above analyses. * = significant at *P* < .01. MASQ-S = Mood and Anxiety Symptom Questionnaire – Short Form.

## Footnotes

1 While data have generally supported the tripartite model, recent evidence indicates that anxious arousal is specifically related to panic and PTSD rather than to anxiety disorders generally. Further, anhedonia is found in social phobia as well as depression (for a review, see Watson, 2005). This, and other evidence, has led to the genesis of a new structural model of emotional disorders, which distinguishes between fear conditions (e.g. panic, social phobia), distress disorders (e.g. GAD, depression, PTSD) and bipolar disorders.

2 To ensure a wide symptom spread we generated two lists of volunteers from our participant panel, one targeting individuals with previous symptom scores in the non-clinical range and one identifying individuals with symptom scores previously in the clinical ranges. The MASQ-S total score across the entire sample was satisfactorily distributed, Kolmogorov–Smirnov test of normality (113) = .08, *P* = .05, skewness = .28, kurtosis = .78, meaning bimodality was not of concern. The sample was not stratified for other demographic variables, such as age, gender or estimated IQ.

3 Following Pollatos et al. (2009), the BDI × STAI-T analysis did not covary general distress or the nuisance variables on previous steps. When these covariates were removed from the Anhedonia × Arousal analysis, the interaction step remained significant, *F*(1,95) = 8.28, *P* < .01, Δ*R^2^* = .06, *t* = 2.87, *r*_p_ = .28, beta = .32.
